# A Case of Type 1 Triallelic Patterns at D5S818, D18S51, D6S1043, and FGA Demonstrated by Short Tandem Repeat Analysis

**DOI:** 10.1155/2022/8600125

**Published:** 2022-04-25

**Authors:** Jing Fan, Ai-Ping Zhang, Zhong-Zheng Zheng, Lin An, Pei-Li Xiao, Dai-Yang Li, Ke-Ming Du, Hao Xiong

**Affiliations:** ^1^Department of Hematology Oncology, Wuhan Children's Hospital (Wuhan Maternal and Child Healthcare Hospital), Tongji Medical College, Huazhong University of Science & Technology, Wuhan 430016, China; ^2^Medical School of Jianghan University, Wuhan Economic and Technological Development Zone, Wuhan 430056, China; ^3^Shanghai Tissuebank Biotechnology Co., Ltd, Shanghai 201201, China

## Abstract

The triallelic pattern of short tandem repeat (STR) is rare; especially, the case where this pattern exists at 4 loci has not been reported. Here, we report the type 1 triallelic patterns at D5S818, D18S51, D6S1043, and FGA from a Chinese family, which were observed during our routine chimerism assays. Before hematopoietic stem cell transplantation, the blood sample of the certain patient was analyzed by performing chimerism analysis. A preliminary STR analysis was also performed on the samples of the patient's parents. STR signal data illustrated that the sum of the peak chart areas of the two types inherited from the father was basically the same as that of the mother, belonging to the type 1 triallelic pattern. In addition, the patient's elder sister's STR result appeared to be normal. Altogether, we presented a pedigree, in which the triallelic pattern was linked by inheritance in the family. This is the first reported case of the triallelic pattern at D5S818, D18S51, D6S1043, and FGA all around the world. We hope that in the future there will be any tools to achieve accurate verification against this possibility.

## 1. Introduction

The short tandem repeat (STR) locus is composed of short, repetitive sequence elements throughout the genomes of a wide range of species [1, 2]. Genotyping of highly polymorphic STR is not only widely used for individual genetic identification in paternity disputes, family relationship testing, and forensic DNA analysis but also for monitoring chimerism after hematopoietic stem cell transplantation (HSCT). Determination of polymorphic STR loci is accepted by the international community using commercial kits and a simple multiplex polymerase chain reaction (PCR) followed by fragment analysis using fluorescent capillary electrophoresis. Chimerism analysis is an important method for monitoring post-HSCT outcomes.

In the diploid autosomal chromosomes of the human genome, two alleles are expected at each STR locus. Regarding homozygotes, two identical alleles present a single peak pattern in capillary electrophoresis analysis, while heterozygotes manifest a two-banded pattern. However, in rare cases, an anomalous pattern of three peaks appears in the electropherogram, that is, the triallelic pattern [3], or the three-banded pattern [4]. In the type 1 pattern, the third allele presents a different peak height from the other two alleles in STR typing and is concerned a result of slippage mutation in early somatic cell development. Here, we report the type 1 triallelic patterns at locus D5S818, D18S51, D6S1043 and FGA from a Chinese family, which were observed during our routine chimerism assays.

## 2. Case Report

The sample carrying the new triallelic patterns was a 9-year-old boy. The four studied subjects including the patient, parents, and elder sister belong to the same family pedigree. All procedures were approved by the Ethics Committee of Shanghai Tissuebank Medical Laboratory, and all individuals volunteered for this study by signing a written informed consent.

All of them had blood samples that were sent to our laboratory for chimerism analysis from Wuhan Children's Hospital Affiliated to Tongji Medical College, Huazhong University of Science & Technology, Wuhan, China. Several hairs were combed naturally from the patient, wrapped with absorbent cotton, and cleaned with detergent. The hairs with hair follicles were cut to about 0.5 cm and placed in a 0.5 ml centrifuge tube. The hairs with hair follicles of about 4–6 cm were cut into sections every 0.5 cm and placed in another 0.5 ml centrifuge tube [5]. DNA was extracted by the QiaAMP® DNA mini kits (QIAGEN GmbH–Hilden, Germany). The AmpFLSTR® Huaxia™ PCR Direct Amplification Kit (Invitrogen Part of Life Technologies, Beijing, China) was used to amplify 15 autosomal STRs and gender marker amelogenin in a 25.0 *μ*l total reaction volume, with a DNA template amount of 0.5–1.25 ng. The amplified products were analyzed using an ABI 3130 genetic analyzer (Applied Biosystems, Foster City, CA, USA) followed by data analysis using GeneMapper v3.2 software. Based on the STRBase website, the specific primer information of locus D5S818, D18S51, D6S1043, and FGA was shown in Table S1. SiFaSTRTM 23-plex system (as previously described [6]) was applied for STR testing to verify the accuracy of the results. Conventional cytogenetic analysis was performed to identify the karyotype of the patient.

**Figure 1:**
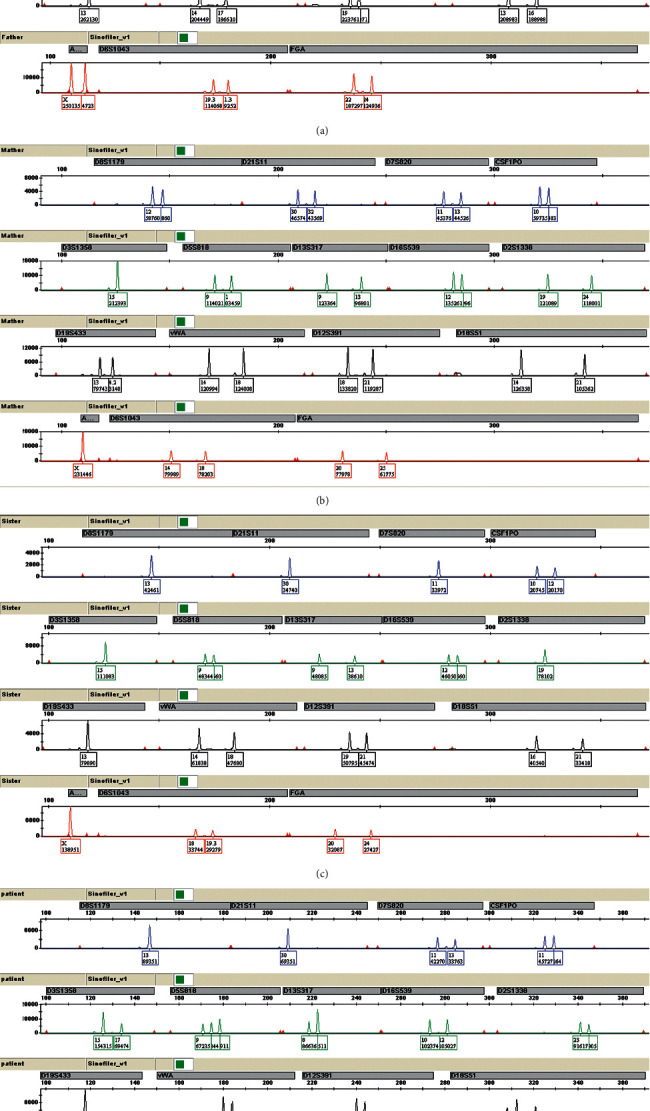
Triallelic patterns at locus D5S818, D18S51, D6S1043, and FGA genotype as depicted by the ABI GeneMapper v3.2 software, including the patient's (a) father, (b) mother, (c) sister, and (d) the patient.

For STR analysis, before hematopoietic stem cell transplantation, the blood sample of this patient was requested to perform chimerism analysis and then a triallelic peak at locus D5S818, D18S51, D6S1043, and FGA was observed. Consistent results were obtained with the SiFaSTRTM 23-plex system (Figure S1). In order to explain this possibility, a preliminary STR analysis was also performed on the samples of the patient's parents. The STR results of the patient and his parents indicated that the four triallelic patterns were all inherited from the father, and the mother's inheritance was normal; furthermore, the STR peak signal data (Figure 1, Table S2) illustrated that the sum of the peak chart areas of the two types inherited from the father was comparable to those of the mother, belonging to the type 1 triallelic pattern. In addition, the patient's elder sister's STR result appeared to be normal.

**Figure 2:**
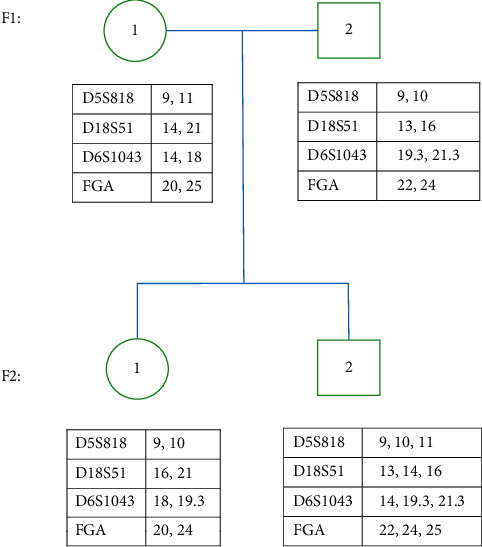
Family pedigree with the DNA profiles of the locus D5S818, D18S51, D6S1043, and FGA.

**Figure 3:**
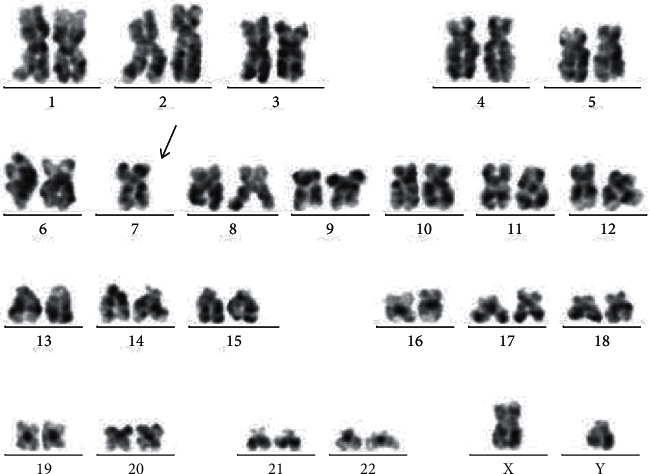
G-banded karyogram of 20 marrow cells at metaphase of the patient. Arrow indicates the missing chromosome 7.

To clarify the genetic mechanism, the DNA profiles of the STR loci were determined by fluorescence-based capillary electrophoresis in the family (Table S3). A total of four types including D5S818, D18S51, D6S1043, and FGA were found to exhibit the triallelic pattern (Figure 2). It is proved that the patient should belong to the chimerism, but it is not yet possible to determine whether inheritance or acquired mutations caused this kind of triallelic pattern based on STR testing alone. Also, conventional cytogenetic analysis of bone marrow cells at the time of diagnosis showed 45, XY, -7(20) (Figure 3).

Interestingly, it was found that the ratio of the two genotypes of the patient's father at the D16S539 locus was basically the same. Taken together, with our study on this pedigree, we speculated that the patient represents an unusual example of tetragametic chimerism. Judging from the peak pattern in EPG and being triallelic both in blood and hair follicle cells, the most plausible explanation is that he is a chimera having cells from an unborn twin in his body.

## 3. Discussion

Triallelic patterns at STR loci can occasionally be observed in routine forensic casework [7]. In view of the fact that there are only single-locus triallelic pattern case reports at present [1, 3, 7–15], but no related cases of multiloci related cases, thus, in order to explore the genetic mechanism, a preliminary STR analysis of the patient's parent sample was performed to clarify the cause of the case. According to the analysis of STR mosaic type and peak value, it could be considered as that of tetragametic. Actually, the most plausible explanation is that he is a chimera having cells from an unborn twin in his body.

During STR typing for individual identification and paternity testing, the triallelic pattern, also named as three-banded pattern, is occasionally observed at a single STR locus on autosomal chromosomes [9]. Triallelic patterns may have multiple causes, such as chimerism or chromosomal mutations. Clayton et al. [8] have distinguished two types of triallelic patterns: type 1, with higher frequency and unbalanced peak size, where the sum of the peak intensity of the affected allele variants is equal to the intensity of the nonmutated alleles; and type 2, where the peaks have balanced intensity. Type 1 triallelic patterns indicate a somatic mutation of an allele at a heterozygous locus during the development of the individual, leading to chimerism. Generally, mutation events are the addition or loss of repetitive units. The triallelic patterns of type 2 indicate an event of duplication located on the same chromosome or translocated or chromosomal aneuploidy (trisomy). When it comes to localized duplication, the two alleles may be inherited together because they will be strongly linked [14]. The probable cause of this chimerism has been outlined in a previous study: separately fertilized XX zygotes, one with HLA haplotypes 1 and 3 and the other with haplotypes 2 and 4, are thought to have fused early in development. The distribution of cell lines varied in individual tissues, except in blood which appeared to be derived from only one cell line, bearing HLA haplotypes 1 and 3 [10]. This mechanism could explain the patterns observed in the reported study.

In addition, this patient has also undergone panel testing by next-generation sequencing, but the structural variation alone cannot be seen (data not shown). It is worth noting that there is currently no precedent for this type of case, and it is still uncertain whether other tests will be required in the future. To this end, although clinical experts recommend three strategies to further verify this possibility, including whole-genome sequencing or third-generation sequencing, and Bionano Optical Mapping [16] to detect structural variations, the effect will be similar to that of STR based on the ratio. In fact, there is currently no means to accurately verify this possibility. After all, the inference based on the data happened very early in development. Besides, given that this patient is still a child (<13 years old), meiotic cells are not yet possible. We hope that in the future there will be tools to achieve accurate verification against this possibility.

In summary, we present a case showing the triallelic patterns at multiple STR loci and discuss their etiologies, which is a rare case and very interesting phenomenon. The triallelic pattern was classified as type 1, and this patient should belong to a mosaic state, but whether the specific cause of this chimerism is inherited or acquired mutation cannot be determined based on STR detection. At the time of writing this article, we found that the existing literature only reported the triallelic pattern of a single locus. This is the first reported case of the triallelic pattern of D5S818, D18S51, D6S1043, and FGA loci worldwide.

## Data Availability

The data generated during and/or analyzed during the current study are available from the corresponding author on reasonable request.

## Supplementary Materials

Supplementary Materials Table S1: specific primer information of locus D5S818, D18S51, D6S1043, and FGA. Table S2: STR types and peak areas at different loci of the patient. Table S3: The DNA profiles of the STR loci tested in the family members. Figure S1: The patient's triallelic patterns at locus D5S818, D18S51, D6S1043, and FGA genotype by the SiFaSTRTM 23-plex system.
