# Supplementary material for: A Case of Type 1 Triallelic Patterns at D5S818, D18S51, D6S1043, and FGA Demonstrated by Short Tandem Repeat Analysis
Source: Int J Clin Pract. 2022 Apr 25;2022:8600125. doi: 10.1155/2022/8600125 (PMC9159177; doi:10.1155/2022/8600125)
Supplement: Supplementary Materials — Table S1: specific primer information of locus D5S818, D18S51, D6S1043, and FGA. Table S2: STR types and peak areas at different loci of the patient. Table S3: The DNA profiles of the STR loci tested in the family members. Figure S1: The patient's triallelic patterns at locus D5S818, D18S51, D6S1043, and FGA genotype by the SiFaSTRTM 23-plex system. [file 8600125.f1.zip › 8600125.f1/Figure S1.docx]

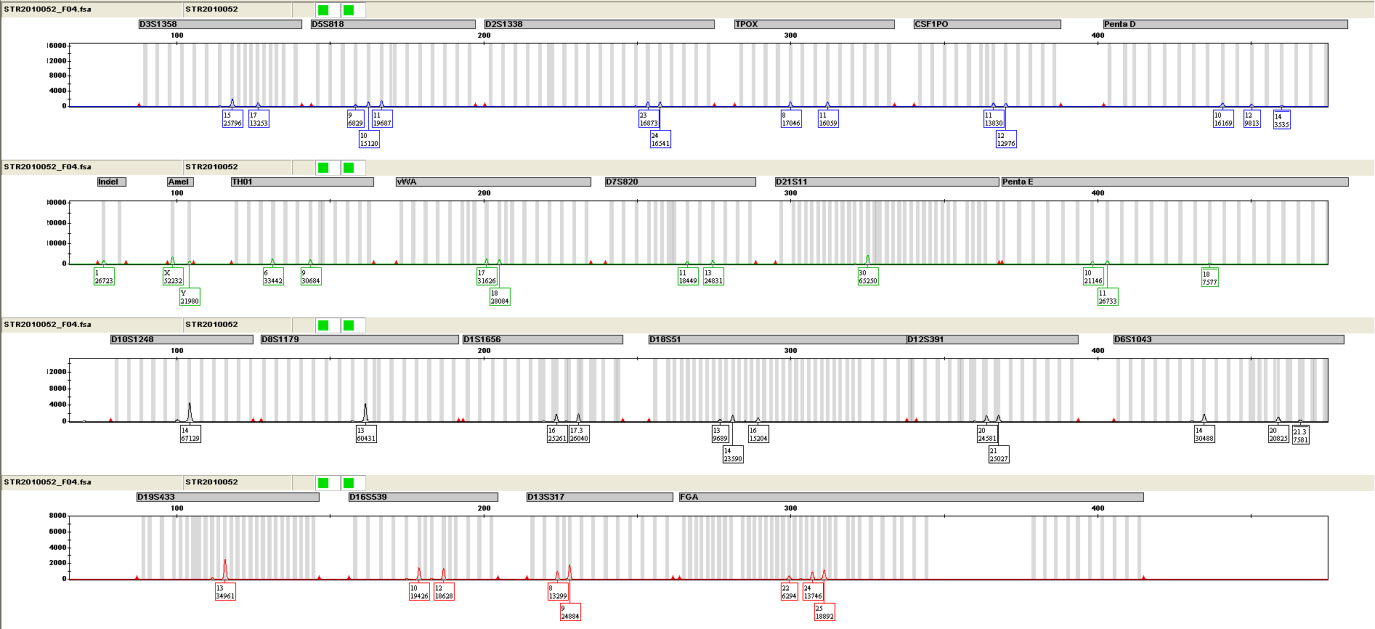


**Figure S1.** The patient's tri-allelic patterns at locus D5S818, D18S51, D6S1043 and FGA genotype by SiFaSTRTM 23-plex system.
