# Supplementary material for: A Case of Type 1 Triallelic Patterns at D5S818, D18S51, D6S1043, and FGA Demonstrated by Short Tandem Repeat Analysis
Source: Int J Clin Pract. 2022 Apr 25;2022:8600125. doi: 10.1155/2022/8600125 (PMC9159177; doi:10.1155/2022/8600125)
Supplement: Supplementary Materials — Table S1: specific primer information of locus D5S818, D18S51, D6S1043, and FGA. Table S2: STR types and peak areas at different loci of the patient. Table S3: The DNA profiles of the STR loci tested in the family members. Figure S1: The patient's triallelic patterns at locus D5S818, D18S51, D6S1043, and FGA genotype by the SiFaSTRTM 23-plex system. [file 8600125.f1.zip › 8600125.f1/Table S1.docx]

**Table S1.** Specific primer information of locus D5S818, D18S51, D6S1043 and FGA.

| **Loci** | **Forward primer** | **Reverse primer** | **Set of primer** | **STRBase website** |
| --- | --- | --- | --- | --- |
| D5S818 | 5'-GGGTGATTTTCCTCTTTGGT-3' | 5'-TGATTCCAATCATAGCCACA-3' | 2 | http://www.cstl.nist.gov/biotech/strbase/str_D5S818.htm |
| D18S51 | 5'-CAAACCCGACTACCAGCAAC-3' (GAAA strand) | 5'-GAGCCATGTTCATGCCACTG-3' | 1 | http://www.cstl.nist.gov/biotech/strbase/str_D18S51.htm |
| D6S1043 | 5'-CAAGGATGGGTGGATCAATA-3' | 5'-TTGTATGAGCCACTTCCCAT-3' | 1 | http://www.cstl.nist.gov/biotech/strbase/str_D6S1043.htm |
| FGA | 5'-GCCCCATAGGTTTTGAACTCA-3' (CTTT strand) | 5'-TGATTTGTCTGTAATTGCCAGC-3' (GAAA strand) | 1 | http://www.cstl.nist.gov/biotech/strbase/str_FGA.htm |
