# Supplementary material for: A Case of Type 1 Triallelic Patterns at D5S818, D18S51, D6S1043, and FGA Demonstrated by Short Tandem Repeat Analysis
Source: Int J Clin Pract. 2022 Apr 25;2022:8600125. doi: 10.1155/2022/8600125 (PMC9159177; doi:10.1155/2022/8600125)
Supplement: Supplementary Materials — Table S1: specific primer information of locus D5S818, D18S51, D6S1043, and FGA. Table S2: STR types and peak areas at different loci of the patient. Table S3: The DNA profiles of the STR loci tested in the family members. Figure S1: The patient's triallelic patterns at locus D5S818, D18S51, D6S1043, and FGA genotype by the SiFaSTRTM 23-plex system. [file 8600125.f1.zip › 8600125.f1/Table S2.docx]

**Table S2.** The STR types and peak area at different locus of the patient.

| **Coordinates of STR locus** | **STR type** | **Peak area** | | |
| --- | --- | --- | --- | --- |
| D8S1179 | 13 | 51803 | \ | \ |
| D21S11 | 30 | 38187 | \ | \ |
| D7S820 | 11、13 | 16956 | 17543 | \ |
| CSF1PO | 11、12 | 6688 | 3510 | \ |
| D3S1358 | 15、17 | 37654 | 14014 | \ |
| D5S818 | 9、10、11 ^a^ | 26683 ^b^ | 37324 ^b^ | 59718 ^c^ |
| D13S317 | 8、9 | 91474 | 202290 | \ |
| D16S539 | 10、12 | 21209 | 18204 | \ |
| D2S1338 | 23、24 | 19988 | 18575 | \ |
| D19S433 | 13 | 49426 | \ | \ |
| vWA | 17、18 | 87602 | 69237 | \ |
| D12S391 | 20、21 | 36753 | 26187 | \ |
| D18S51 | 13、14、16 ^a^ | 35926 ^b^ | 68230 ^c^ | 40356 ^b^ |
| AMEL | X、Y | 151176 | 82776 | \ |
| D6S1043 | 14、19.3、21.3 ^a^ | 50876 ^c^ | 23071 ^b^ | 24707 ^b^ |
| FGA | 22、24、25 ^a^ | 20701 ^b^ | 29329 ^b^ | 39291 ^c^ |

^a^ The STR loci with a tri-allelic pattern of the patient.

^b^ The type comes from the patient’s father.

^c^ The type comes from the patient’s mather.
