# Supplementary material for: A Case of Type 1 Triallelic Patterns at D5S818, D18S51, D6S1043, and FGA Demonstrated by Short Tandem Repeat Analysis
Source: Int J Clin Pract. 2022 Apr 25;2022:8600125. doi: 10.1155/2022/8600125 (PMC9159177; doi:10.1155/2022/8600125)
Supplement: Supplementary Materials — Table S1: specific primer information of locus D5S818, D18S51, D6S1043, and FGA. Table S2: STR types and peak areas at different loci of the patient. Table S3: The DNA profiles of the STR loci tested in the family members. Figure S1: The patient's triallelic patterns at locus D5S818, D18S51, D6S1043, and FGA genotype by the SiFaSTRTM 23-plex system. [file 8600125.f1.zip › 8600125.f1/Table S3.docx]

**Table S3.**  The DNA profiles of the STR loci tested in the family members.

| **STR loci** | **Family members** | | | |
| --- | --- | --- | --- | --- |
|  | **F1-1** | **F1-2** | **F2-1** | **F2-2** |
| D8S1179 | 13 | 12, 13 | 13 | 13 |
| D21S11 | 30 | 30, 32 | 30 | 30 |
| D7S820 | 11, 12 | 11, 13 | 11 | 11, 13 |
| CSF1PO | 12 | 10, 11 | 10, 12 | 11, 12 |
| D3S1358 | 15, 17 | 15 | 15 | 15, 17 |
| D5S818 | 9, 10 | 9, 11 | 9, 10 | 9, 10, 11 |
| D13S317 | 8, 9 | 9, 13 | 9, 13 | 8, 9 |
| D16S539 | 10, 12 | 12, 13 | 12, 13 | 10, 12 |
| D2S1338 | 19, 23 | 19, 24 | 19 | 23, 24 |
| D19S433 | 13 | 13, 14.2 | 13 | 13 |
| vWA | 14, 17 | 14, 18 | 14, 18 | 17, 18 |
| D12S391 | 19, 20 | 18, 21 | 19, 21 | 20, 21 |
| D18S51 | 13, 16 | 14, 21 | 16, 21 | 13, 14, 16 |
| AMEL | X, Y | X | X | X, Y |
| D6S1043 | 19.3, 21.3 | 14, 18 | 18, 19.3 | 14, 19.3, 21.3 |
| FGA | 22, 24 | 20, 25 | 20, 24 | 22, 24, 25 |
